# Supplementary material for: Structural insights into the contactin 1 – neurofascin 155 adhesion complex
Source: Nat Commun. 2022 Nov 3;13:6607. doi: 10.1038/s41467-022-34302-9 (PMC9633819; doi:10.1038/s41467-022-34302-9)
Supplement: Supplementary file 1 — Supplementary Information [file 41467_2022_34302_MOESM1_ESM.pdf]

Structural insights into the contactin 1 – neurofascin 155 adhesion complex

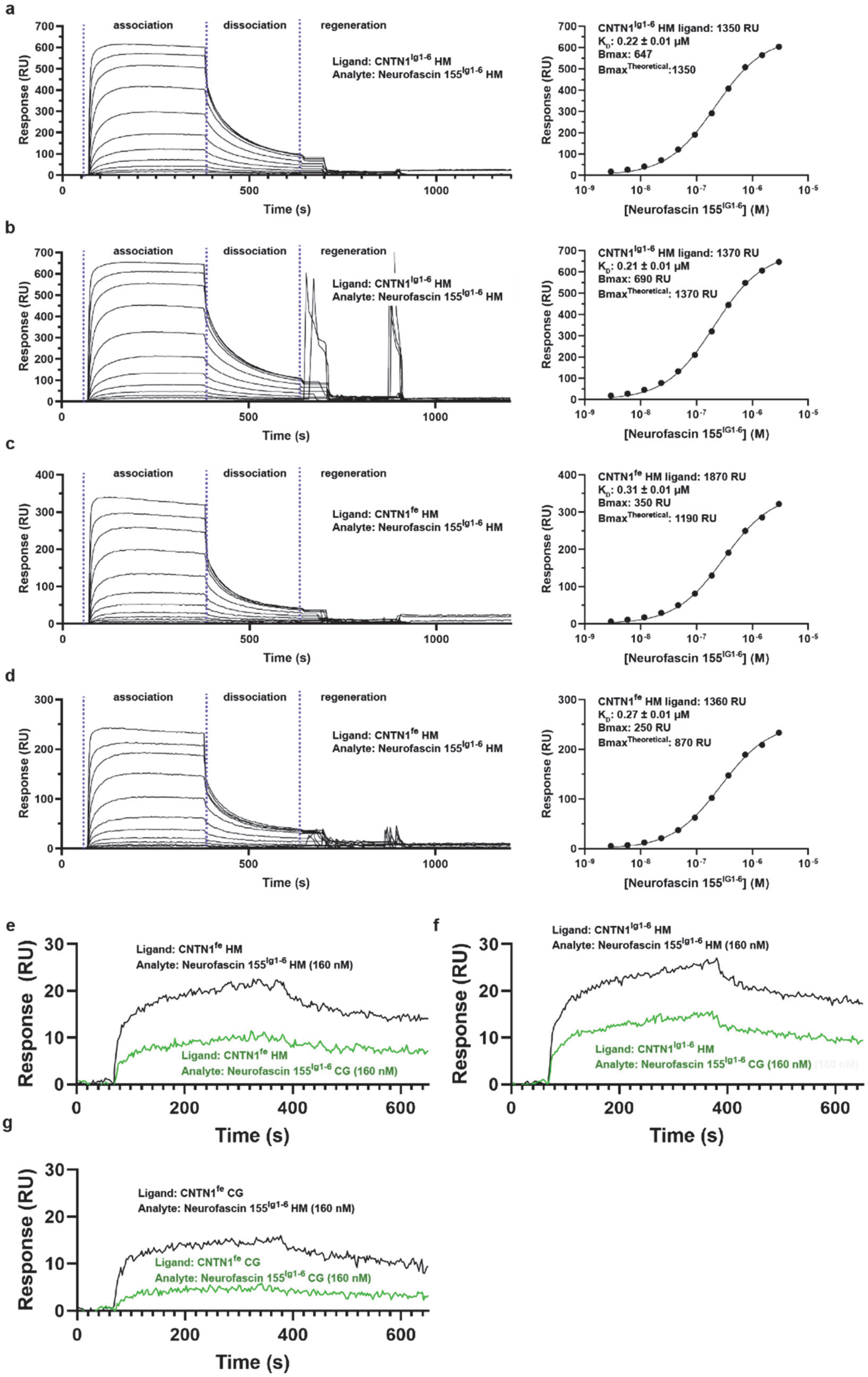

Supplementary Figure 1: Contactin 1 and neurofascin 155 interact. **a-d** Surface plasmon resonance imaging interaction data using spotted protein arrays of contactin 1^{fe} HM and contactin 1$^{Ig1\text{-}6}$ HM ligands with neurofascin 155$^{Ig1\text{-}6}$ HM analyte. a-b and c-d are technical duplicates, i.e independent positions on the sensor surface. SPR sensorgrams, with the association, dissociation and regeneration phases indicated are shown left. Equilibrium binding data versus analyte concentration modeled with a 1:1 Langmuir binding model are shown right. The theoretical Bmax is calculated as the amount of deposited ligand on the surface corrected for the difference in molecular weight between the ligand and the analyte and assuming a 1:1 interaction. **e-g** Qualitative comparison of neurofascin 155$^{Ig1\text{-}6}$ HM (black lines) and CG (green lines) versions binding to contactin 1^{fe} HM (**e**) and CG (**g**), and contactin 1$^{Ig1\text{-}6}$ HM (**f**) ligands. The lower response of neurofascin 155$^{Ig1\text{-}6}$ CG compared to neurofascin 155$^{Ig1\text{-}6}$ HM at equivalent concentrations indicates the CG version of neurofascin 155$^{Ig1\text{-}6}$ binds with lower affinity to contactin 1 compared to the neurofascin 155$^{Ig1\text{-}6}$ HM version. Contactin 1 (CNTN1), domains Ig1 to Ig6 (Ig1-6), full ectodomain (fe), high mannose glycans (HM), complex glycans (CG), maximum analyte binding (B_{max}), response units (RU). Source data are provided as a Source Data file.

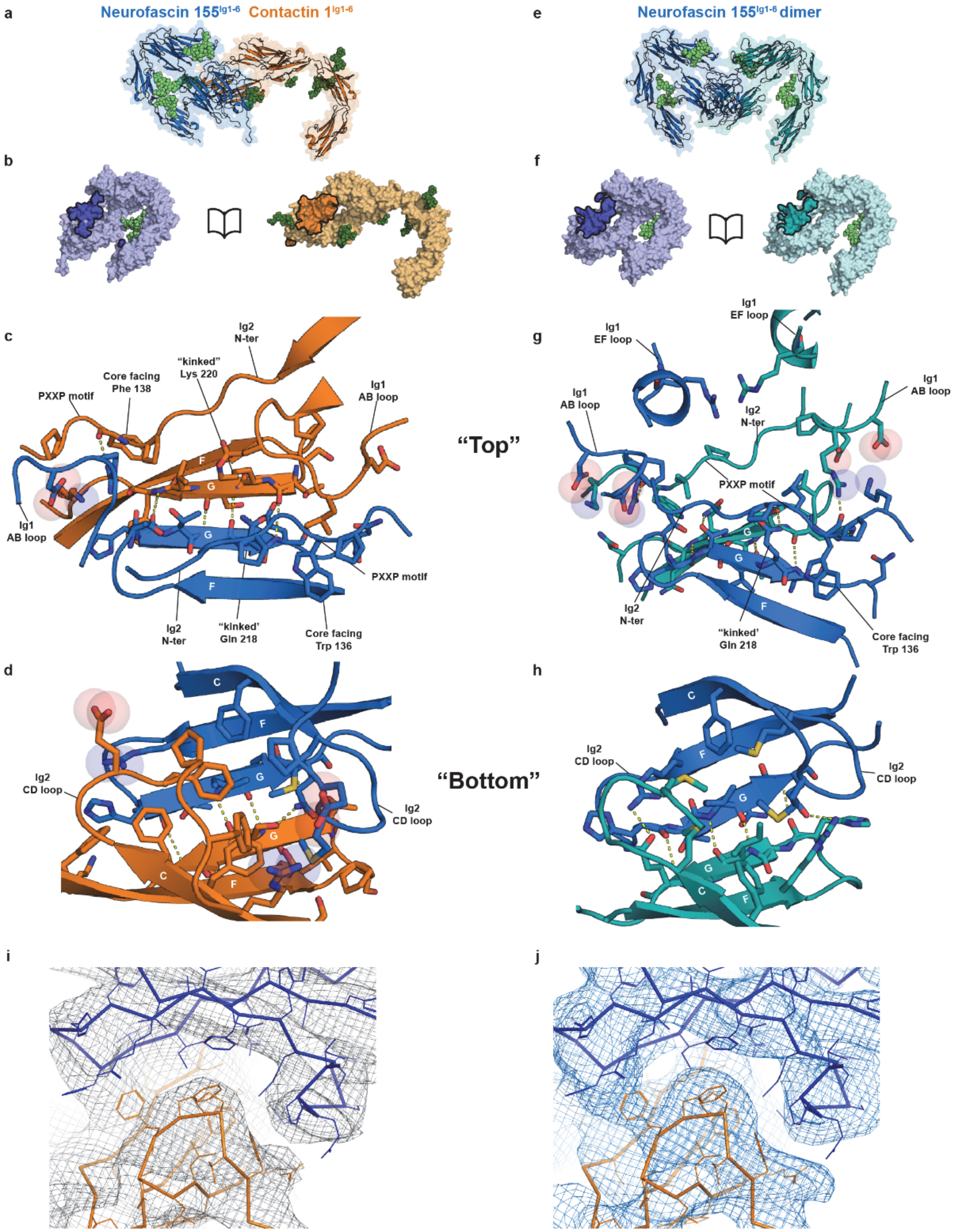

Supplementary Figure 2: Comparison of contactin 1^{Ig1-6} – neurofascin 155^{Ig1-6} and neurofascin 155^{Ig1-6} dimer Ig1-2 mediated interaction interfaces. **a-d** Contactin 1^{Ig1-6} – neurofascin 155^{Ig1-6} complex. **b** Open book representation of contactin 1^{Ig1-6} – neurofascin 155^{Ig1-6} complex in surface representation with interaction surfaces outlined. **c-d** Residues at the "top" (**c**) and "bottom" (**d**) of the GFC super beta sheet involved in interface formation. **e-f** Neurofascin 155^{Ig1-6} dimer. **f** Open book representation of neurofascin 155^{Ig1-6} dimer molecules in surface representation with interaction surfaces outlined. **g-h** Dimer residues at the "top" (**g**) and "bottom" (**h**) of the GFC super beta sheet involved in dimerization interface formation. **i** Contactin 1 - neurofascin 155 interface electron density ($2mF_{obs}$-DF_{calc}) at 1σ level and B-factor sharpened by -150 Å^2 of a view similar to that shown in fig. 1d right-bottom panel. **j** Same view as in **(i)** showing the interface omit electron density ($2mF_{obs}$-DF_{calc}) from a composite omit map at 1σ level, not B-factor sharpened and indicating the model bias in the contactin 1^{Ig1-6} – neurofascin 155^{Ig1-6} complex is minimal.

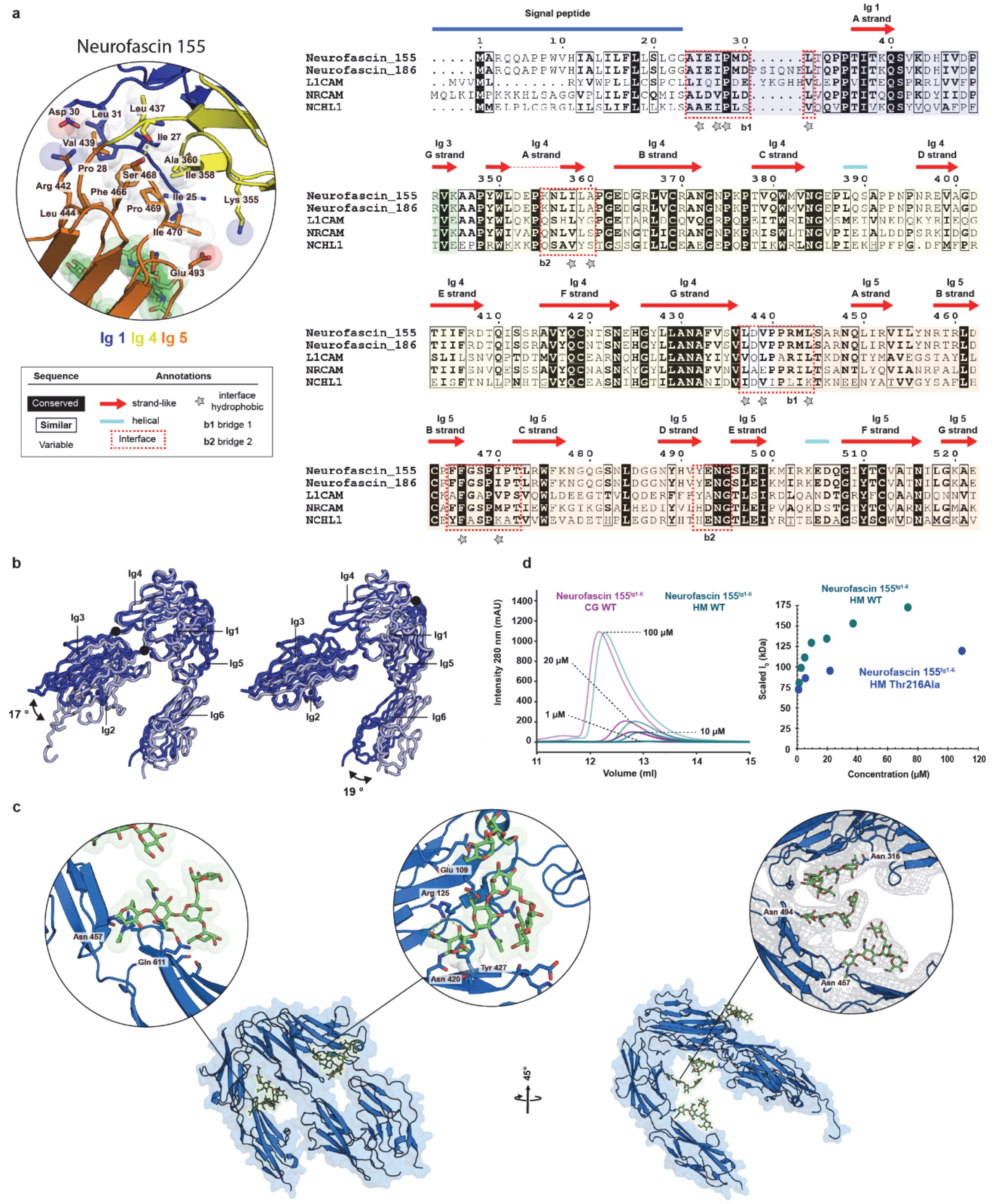

Supplementary Figure 3: Characteristic architecture, flexibility and glycosylation of the neurofascin 155^{Ig1-6} monomer. **a** N-terminus and Ig 1-4-5 neurofascin 155^{Ig1-6} interface L1 family conservation analysis. **b** Superposed neurofascin 155^{Ig1-6} chains showing 17° Ig2-Ig3 hinging with respect to Ig 1 and 4, and 19° Ig5-Ig6 hinging with respect to Ig 4. **c** (Left) neurofascin 155^{Ig1-6} dimer dataset chain A illustrating contacts made between N-linked glycans and adjacent domains. (Right) neurofascin 155^{Ig1-6} complex dataset chain D with glycan electron density (2mF$_{obs}$-DF$_{calc}$) at 1σ shown as grey mesh illustrating the close proximity of the glycans. **d** (Left) Neurofascin 155^{Ig1-6} wt HM and CG SEC analysis. (Right) Neurofascin 155^{Ig1-6} HM wt and Thr216Ala I_0 vs concentration. High mannose glycans (HM), complex glycans (CG), milli absorbance units (mAU). Source data are provided as a Source Data file.

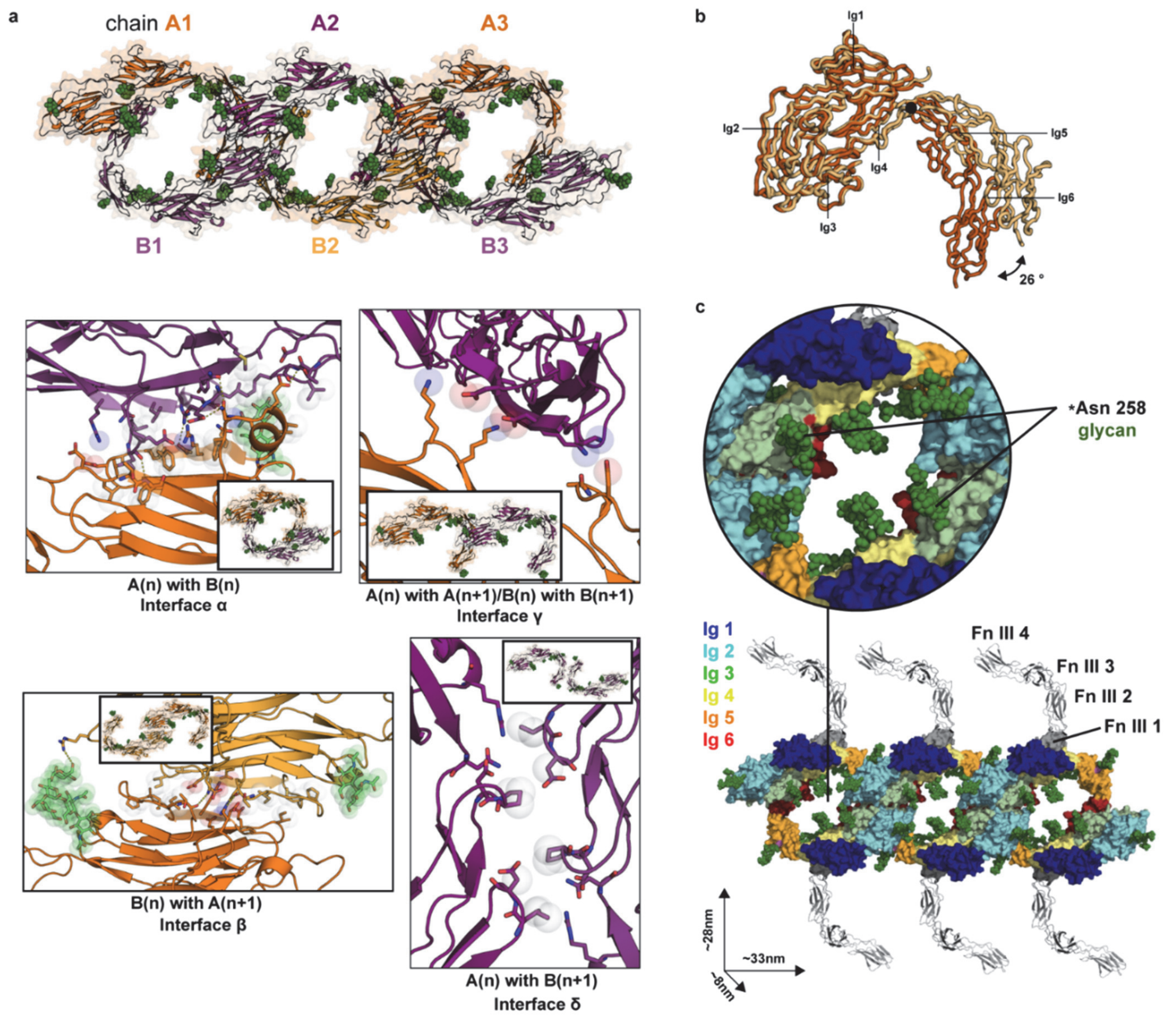

Candidate species	Interface	Sum of Contributions	jsPISA buried surface Area Å²
A(n), B(n) dimer	2α	2980	2980
A(n+1), B(n) dimer	β	1190	1190
A(n), A(n+1) dimer	γ	420	420
B(n), B(n+1) dimer	γ	460	460
A(n), B(n+1) dimer	δ	340	340
A1, B1, A2 trimer	2α+β+γ	2980 + 1190 + 420	4590
A1, B1, B2 trimer	2α+γ+δ	2980 + 460 + 340	3780
A1, B1, A2, B2 tetramer	4α+β+2γ+δ	2980 + 2980 + 1190 + 420 + 460 + 340	8370

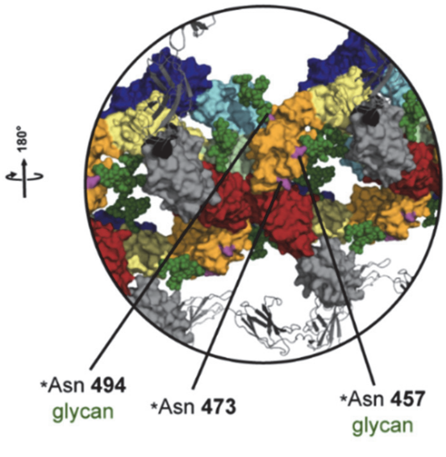

Supplementary Figure 4: Contactin 1^{Ig1-6} zipper features and flexibility. **a** Contactin 1^{Ig1-6} zipper with chains colored by chain (top). Corresponding zipper interfaces (α-δ) showing residue level contributions (middle). Buried surface areas of the individual interfaces and their combinations in the zipper (bottom). **b** Superposed contactin 1^{Ig1-6} chains showing 26° hinging in the Ig4-Ig5 connection. **c** Contactin 1^{fe} model superposed on the contactin 1^{Ig1-6} zipper. Immunoglobulin domains are rainbow colored while fibronectin domains in grey are annotated. Asparagines affecting neurofascin binding in cellular assays and targeted by neuropathies as shown previously[45] are colored pink in the bottom panel.

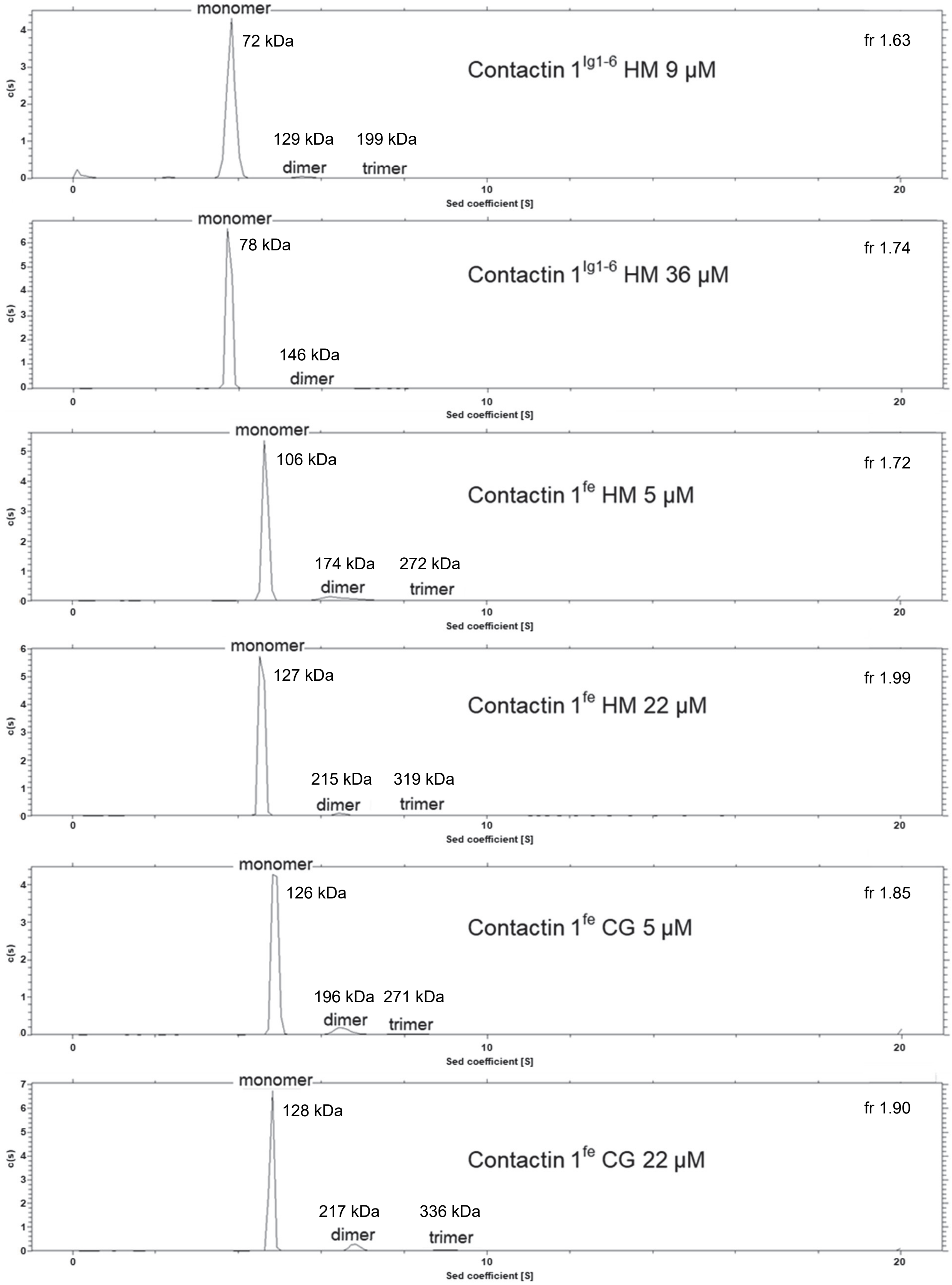

Supplementary Figure 5: Continued on the next page.

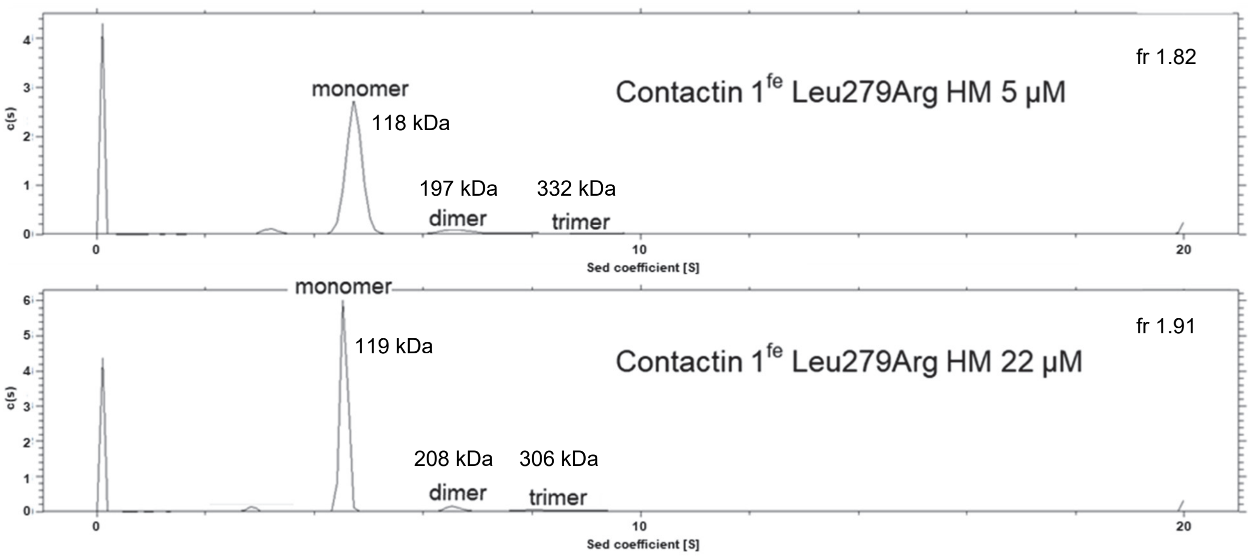

Supplementary Figure 5 (continued): Analytical ultracentrifugation (AUC) fits for sedimentation velocity data of contactin 1 variants at different concentrations. The peaks in the AUC were assigned as monomer, dimer and trimer based on the S values and the molecular weight calculated using a weight-average best-fit frictional ratio. The molecular weight (Mw), based on the peak S value, and best-fit frictional ratio (fr) are mentioned in each experimental panel. High mannose glycans (HM), complex glycans (CG), sedimentation coefficient distribution c(s). Source data are provided as a Source Data file.

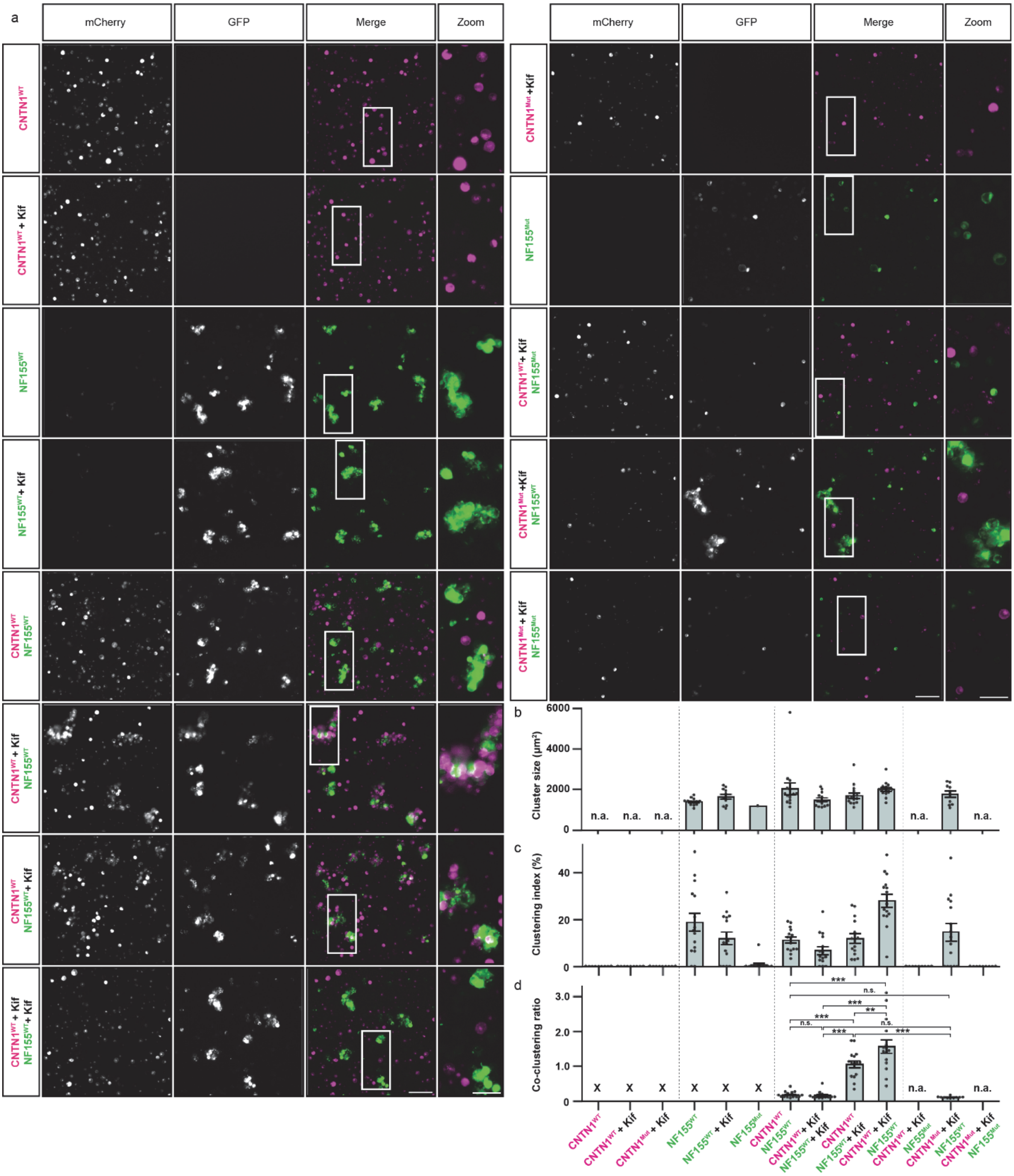

Supplementary Figure 6: Contactin 1 – neurofascin 155 expression mediates cell clustering in K562 cells. **a** Representative cell clustering images of K562 cells expression contactin 1 (mCherry; magenta; CNTN1) and neurofascin 155 (GFP; green; NF155). Image labels indicate the proteins being expressed and the presence of kifunensine (+Kif; 10 μM) during the expression of contactin 1 or neurofascin 155. Each experiment was repeated three times independently with similar results. Insets indicate zoom images on the right. Scale bar, 100 μm and 50 μm in zoomed images. **b** Cluster area size in μm^2. **c** Clustering index; the proportion of the total segmented cell area classified as a cluster. **d** Co-clustering ratio determined as the mean mCherry signal divided by the mean GFP signal per cluster. Single data points in **b-d** represent the average values from the n = 15 images (n = 14 for neurofascin 155^{mut} and contactin 1^{Mut} – neurofascin 155^{Mut}) from N = 3 independent experiments (each experiment 5 images). Error bars indicate the mean ± SEM. Statistical significance was determined by performing a one-way ANOVA followed by a Tukey's multiple comparison test, and results are indicated using the following conventions: n.s.: not significant, **: $p < 0.01$, ***: $p < 0.001$. In absence of clusters for analysis 'n.a' is indicated, and 'X' denote single-channel conditions in which no fluorescence ratio could be calculated. P values are as follows: >0.9999 CNTN1/NF155 vs. CNTN1+Kif/NF155+Kif, <0.0001 CNTN1/NF155 vs. CNTN1/NF155+Kif, <0.0001

CNTN1/NF155 vs. CNTN1+Kif/NF155, 0.9911 CNTN1/NF155 vs. CNTN1mut+Kif/NF155, <0.0001 CNTN1+Kif/NF155+Kif vs. CNTN1/NF155+Kif, <0.0001 CNTN1+Kif/NF155+Kif vs. CNTN1+Kif/NF155, 0.998 CNTN1+Kif/NF155+Kif vs. CNTN1mut+Kif/NF155 vs. CNTN1mut/NF155, 0.0099 CNTN1/NF155+Kif vs. CNTN1+Kif/NF155, <0.0001 CNTN1/NF155+Kif vs. CNTN1mut+Kif/NF155. Source data are provided as a Source Data file.

Supplementary Table 1: Neurofascin 155^{Ig1-6} analytical gel filtration and MALS data summary.

Protein	Conc. at injection (µM)	Glycan	Expected Monomer Mw (kDa)	Peak retention volume (mL)	MALS Mw (kDa)
Neurofascin^{Ig1-6}	1	High mannose	76	13.16	80.5 ± 4.3
	10	High mannose	76	13.03	81.9 ± 0.33
	20	High mannose	76	12.86	86.3 ± 0.35
	100	High mannose	76	12.37	102.9 ± 0.41
Neurofascin^{Ig1-6} T216A	1	High mannose	76	13.30	76.2 ± 2.4
	10	High mannose	76	13.27	76.3 ± 0.46
	20	High mannose	76	13.17	77.4 ± 0.33
	100	High mannose	76	13.24	77.4 ± 0.39

Supplementary Table 2: Batch small-angle X-ray scattering data summary.

Construct	Conc. (mg/ml)	Conc. (μM)	Glycan	Expected Monomer Mw (kDa)	Guinier I_0	Guinier Mw from I_0 (kDa)	Guinier Rg (nm)	Guinier Oligomerization state	P_r d_{max} (nm)
Neurofascin^{Ig1-6}	0.09	1.3	High mannose	76	0.006	81	3.90 ± 0.72	1.1	12.6
	0.19	2.7	High mannose	76	0.014	99	4.06 ± 0.31	1.3	n.d
	0.36	5.1	High mannose	76	0.030	112	4.17 ± 0.18	1.5	n.d
	0.66	9.5	High mannose	76	0.065	129	4.30 ± 0.10	1.7	n.d
	1.38	19.7	High mannose	76	0.140	135	4.49 ± 0.06	1.8	n.d
	2.59	37.2	High mannose	76	0.300	153	4.68 ± 0.06	2.0	n.d
	5.13	73.5	High mannose	76	0.670	173	4.98 ± 0.05	2.3	n.d
Neurofascin^{Ig1-6} T216A	0.07	1.1	High mannose	76	0.004	72	3.58 ± 3.17	1.0	10.6
	0.38	5.5	High mannose	76	0.025	86	3.81 ± 0.64	1.1	n.d
	1.52	21.9	High mannose	76	0.110	95	3.90 ± 0.20	1.3	n.d
	7.62	109.3	High mannose	76	0.690	120	4.29 ± 0.14	1.6	n.d
Contactin 1^{Ig1-6}	0.18	2.7	High mannose	76	0.012	89	4.87 ± 0.80	1.1	14.5
	0.36	5.5	High mannose	76	0.025	93	4.99 ± 0.39	1.2	n.d
	0.73	10.9	High mannose	76	0.055	102	5.04 ± 0.21	1.3	n.d
	1.46	21.8	High mannose	76	0.120	111	5.20 ± 0.19	1.4	n.d
Contactin 1^{Ig1-6}	0.21	3.1	Complex glycan	79	0.018	118	4.90 ± 0.64	1.5	15.8
	0.41	6.2	Complex glycan	79	0.041	135	4.86 ± 0.25	1.7	n.d
	0.82	12.3	Complex glycan	79	0.086	142	4.94 ± 0.14	1.8	n.d
	1.65	24.6	Complex glycan	79	0.170	140	4.96 ± 0.14	1.8	n.d
Contactin 1^{fe}	0.26	2.4	High mannose	119	0.032	164	6.77 ± 1.07	1.3	19.5
	0.60	5.5	High mannose	119	0.068	151	6.83 ± 0.34	1.2	n.d
	1.22	11.1	High mannose	119	0.140	153	6.79 ± 0.19	1.3	n.d
	2.26	20.5	High mannose	119	0.260	154	7.00 ± 0.23	1.3	n.d
Contactin 1^{fe}	0.27	2.5	Complex glycan	122	0.040	195	7.96 ± 0.83	1.6	25.7
	0.55	5.0	Complex glycan	122	0.086	209	7.96 ± 0.52	1.7	n.d
	1.10	10.0	Complex glycan	122	0.170	207	7.96 ± 0.27	1.7	n.d
	2.20	20.0	Complex glycan	122	0.360	219	8.12 ± 0.28	1.8	n.d

Models	Glycan	Oligomerization state	Rg (nm)
Neurofascin^{Ig1-6}	Mannose modelled	Monomer	3.43
Neurofascin^{Ig1-6}	Mannose modelled	Dimer	4.59
Contactin1^{Ig1-6}	Mannose modelled	Monomer	4.25
Contactin1^{fe}	Mannose modelled	Monomer	6.79

Supplementary Table 3: Primers used for DNA amplification and mutagenesis.

Type	protein	Res #	Mut	Restr.	Sequence
forward	Neurofascin 155	25	N/A	BamHI	AATAATGGATCCATCGAGATCCCCATGGATCTG
reverse	Neurofascin 155 (Ig1-6)	633	N/A	NotI	AATAATGCGGCCGCAGCGGCCAGTCTATTGGTAGG
reverse	Neurofascin 155 (fe)	1059	N/A	NotI	AATAATGCGGCCGCCCAGCCTTGTGTGGCAATGTC
forward	Contactin 1	21	N/A	BamHI	AATAATGGATCCGACTTTACCTGGCACAGAAGATATG
reverse	Contactin 1 (Ig1-6)	604	N/A	NotI	AATAATGCGGCCGCGCCTCGAACGACAAGGTCAG
reverse	Contactin 1 (fe)	996	N/A	NotI	AATAATGCGGCCGCGCCTGAAATTTTGACTTGAGACAC
forward	Neurofascin 155	N/A	Thr216Ala	N/A	CTTCACCCACGCCATCCAGCAGAAG
reverse	Neurofascin 155	N/A	Thr216Ala	N/A	TGGAACCGGGCGTTGCAG
forward	Neurofascin 155	N/A	Phe168Asp, Met170Asp, Met174Asp	N/A	CAGCAGCGATGAACCCATCACACAGGAC
reverse	Neurofascin 155	N/A	Phe168Asp, Met170Asp, Met174Asp	N/A	CTATCCCAATCGATCACTGGAGAAGGCAG
forward	Neurofascin 155	N/A	Ile217Asp	N/A	CACCCACACCGATCAGCAGAAGAACCCC
reverse	Neurofascin 155	N/A	Ile217Asp	N/A	AAGTGGAACCGGGCGTTG
forward	Contactin 1	N/A	Leu279Arg	N/A	GCGGAAGGTGCGAGAACCAATGC
reverse	Contactin 1	N/A	Leu279Arg	N/A	CACCGGATATCAGGAACAGG
forward	Contactin 1	N/A	Phe177Asp, Phe180Asp	N/A	GTAGATATCACCATGGATAAGCGAAG
reverse	Contactin 1	N/A	Phe177Asp, Phe180Asp	N/A	AGGATCTTCATTCAGAAGCCAGCG
forward	Contactin 1	N/A	Phe212Asp	N/A	CTACTCCTGCGATGTGTCCAGTC
reverse	Contactin 1	N/A	Phe212Asp	N/A	TTGCCTCTGTCAGAAGAC

Supplementary Table 4: Neurofascin 155 codon optimized DNA.

```
GGATCCGCCACCATGGCTAGACAACAAGCTCCTCCTTGGGTGCACATTGCCCTGATCCTGTTTCTGCTGTCTCTCGGCGGAGCCA
TCGAGATCCCCATGGATCTGACACAGCCTCCAACCATCACCAAGCAGAGCGTGAAGGACCACATCGTGGACCCCAGAGACAACAT
CCTGATCGAGTGCGAGGCCAAGGGCAACCCTGCTCCTAGCTTTCACTGGACCCGGAACAGCCGGTTCTTCAATATCGCCAAAGAT
CCCCGGGTGTCCATGAGAAGGCGCTCTGGCACACTGGTCATCGACTTCAGATCTGGCGGCAGACCCGAGGAATACGAGGGCGAGT
ATCAGTGCTTCGCCCGGAACAAGTTTGGCACAGCCCTGAGCAACCGGATCAGACTGCAGGTTTCCAAGTCTCCCCTGTGGCCTAA
AGAAAATCTGGACCCCGTGGTGGTGCAAGAGGGCGCTCCACTGACTCTGCAGTGCAATCCTCCACCTGGCCTGCCTTCTCCAGTG
ATCTTCTGGATGAGCAGCAGCATGGAACCCATCACACAGGACAAGAGAGTGTCCCAGGGCCACAACGGCGACCTGTACTTCAGCA
ACGTGATGCTGCAGGACATGCAGACCGACTACAGCTGCAACGCCCGGTTCCACTTCACCCACACCATCCAGCAGAAGAACCCCTT
CACACTGAAGGTGCTGACCAACAATCCCTACAACGACAGCAGCCTGCGGAATCACCCCGACATCTATTCTGCTAGAGGCGTGGCC
GAGCGGACCCCTAGCTTTATGTATCCTCAAGGCACCAGCAGCAGCCAGATGGTGCTGAGAGGAATGGACCTGCTGCTCGAGTGTA
TCGCCAGCGGAGTGCCCACACCTGATATCGCCTGGTACAAGAAAGGCGGCGATCTGCCCAGCAACAAGGCCAAGTTCGAGAACTT
TAACAAGGCCCTGAGGATCACCAACGTGTCCGAAGAGGATAGCGGCGAGTACTTCTGCCTGGCCTCCAACAAGATGGGCAGCATC
AGACACACCATCAGCGTCAGAGTGAAGGCCGCTCCTTACTGGCTGGACGAGCCCAAGAACCTGATTCTGGCCCCTGGCGAAGATG
GCAGACTCGTGTGTAGAGCCAACGGCAATCCCAAGCCTACCGTGCAGTGGATGGTCAACGGCGAGCCTCTGCAAAGCGCCCCTCC
TAATCCTAACAGAGAGGTGGCCGGCGACACCATCATCTTCAGGGATACCCAGATCAGCAGCAGAGCCGTGTACCAGTGCAACACC
AGCAACGAGCACGGCTACCTGCTGGCCAATGCCTTTGTGTCCGTGCTGGACGTGCCACCTAGAATGCTGAGCGCCAGAAACCAGC
TGATCAGAGTGATCCTGTACAACCGGACCAGGCTGGACTGCCCATTCTTCGGCAGCCCCATTCCTACACTGCGGTGGTTCAAGAA
TGGCCAGGGCAGCAATCTGGACGGCGGCAACTATCACGTGTACGAGAACGGCAGCCTGGAAATCAAGATGATCCGGAAAGAGGAC
CAGGGCATCTACACCTGTGTGGCCACCAATATCCTGGGCAAAGCCGAGAATCAAGTGCGGCTGGAAGTGAAAGACCCCACCAGAA
TCTACAGAATGCCCGAGGATCAGGTGGCCAAGAGGGGCACAACAGTGCAGCTGGAATGTAGAGTGAAGCACGACCCCAGCCTGAA
GCTGACCGTGTCCTGGCTGAAGGACGATGAGCCTCTGTACATCGGCAACCGCATGAAGAAAGAGGATGACAGCCTGACCATCTTC
GGAGTGGCCGAGAGAGATCAGGGCTCCTACACATGCATGGCCAGCACCGAGCTGGATCAGGATCTGGCCAAGGCCTACCTGACAG
TGCTGGCCGATCAGGCCACACCTACCAATAGACTGGCCGCTCTGCCTAAGGGCAGACCTGACAGACCCAGAGATCTGGAACTGAC
CGACCTGGCCGAAAGATCCGTCCGGCTGACATGGATTCCCGGCGACGACAACAACAGCCCCATCACCGATTACGTGGTGCAGTTC
GAGGAAGATCAGTTTCAGCCCGGCGTGTGGCACGACCACTCTAGATTCCCTGGCTCCGTGAATAGCGCCGTGCTGCACCTGTCTC
CATACGTGAACTACCAGTTTAGAGTGATCGCCGTGAACGAAGTGGGCAGCTCTCACCCTAGCCTGCCTAGCGAGAGATACAGAAC
AAGCGGAGCCCCTCCTGAGAGCAACCCCTCTGATGTGAAAGGCGAGGGCACCAGAAAGAACAACATGGAAATCACCTGGACACCC
ATGAACGCCACCAGCGCCTTCGGACCTAATCTGCGGTACATCGTGAAGTGGCGGCGGAGAGAGACACGGGAAACCTGGAACAATG
TGACCGTGTGGGGCAGCAGATACGTCGTGGGACAGACACCAGTGTACGTGCCCTACGAGATTAGAGTGCAGGCCGAGAACGACTT
CGGCAAGGGACCTGAGCCTGATACCATCATCGGCTACAGCGGCGAGGACTACCCTAGAGCCGCTCCTACCGAAGTGAAGATCCGG
GTGCTGAACAGCACCGCCATTAGCCTGCAGTGGAACAGAGTGTACAGCGACACAGTGCAGGGACAGCTGAGAGAGTACCGGGCCT
ACTATTGGAGAGAGTCTAGCCTGCTGAAGAACCTGTGGGTGTCCCAGAAGAGGCAGCAGGCCTCTTTTCCTGGCGATCGACCAAG
AGGCGTCGTGGCCAGACTGTTCCCCTACTCCAACTACAAGCTGGAAATGGTGGTCGTGAACGGCAGAGGCGACGGCCCTAGAAGC
GAGACAAAAGAGTTCACAACCCCTGAGGGCGTGCCCAGCGCTCCTAGAAGATTCAGAGTGCGGCAGCCCAACCTGGAAACCATCA
ATCTGGAATGGGATCACCCTGAGCACCCCAACGGCATCCTGATTGGCTACATCCTGAGATACGTGCCATTCAACGGCACCAAGCT
GGGCAAGCAGATGGTGGAAAACTTCAGCCCCAACCAGACCAAGTTCAGCGTGCAGAGAGCCGATCCAGTCTCCAGATACCGGTTC
AGCCTGAGCGCTAGAACCCAAGTTGGATCTGGCGAGGCCGCCACAGAGGAATCTCCAGCTCCTCCAAATGAGGCTACCCCTACCG
CCGCCTACACAAACAACCAGGCCGACATTGCCACACAAGGCTGGTTCATCGGCCTGATGTGTGCTATCGCCCTGCTCGTGCTGAT
CCTGCTGATCGTGTGCTTCATCAAGAGAAGCAGAGGCGGCAAGTACCCCGTGCGCGAGAAGAAAGATGTGCCTCTGGGACCAGAG
GACCCCAAAGAAGAGGACGGCAGCTTCGACTACTCCGACGAGGATAACAAACCTCTGCAGGGCAGCCAGACCAGCCTGGATGGAA
CCATCAAGCAGCAAGAGTCCGACGACAGCCTGGTGGATTATGGCGAAGGCGGAGAGGGCCAGTTCAACGAGGATGGCTCTTTTAT
CGGCCAGTACACCGTGAAGAAGGACAAAGAGGAAACCGAGGGCAACGAGAGCAGCGAGGCTACATCTCCCGTGAACGCCATCTAC
AGCCTGGCTGCGGCCGC
```

Supplementary Table 5: Statistics of the cell clustering experiments.

**Metric**	**Clustering index**
**Type of tests**	One-way ANOVA on selected data
	Tukey multiple comparison test
**Related figures**	5c
**ANOVA statistics**	F = 14.88<br>p = <0.0001
**Degrees of freedom**	147
**Tukey's multiple comparison test result (clustering index)**	**Adjusted p-value**
NF155 vs. CNTN1mut+Kif/NF155	0.9544
CNTN1+Kif/NF155 vs. CNTN1+Kif/NF155mut	<0.0001
CNTN1+Kif/NF155 vs. CNTN1mut+Kif/NF155	0.0003
CNTN1+Kif/NF155 vs. CNTN1mut+Kif/NF155mut	<0.0001
**Metric**	**Co-clustering ratio**
**Type of tests**	One-way ANOVA on all data
	Tukey multiple comparison test
**Related figures**	5d, suppl. 6d
**ANOVA statistics**	F = 35.68<br>p = <0.0001
**Degrees of freedom**	66
**Tukey's multiple comparison test result (co-clustering ratio)**	**Adjusted p-value**
CNTN1/NF155 vs. CNTN1+Kif/NF155+Kif	>0.9999
CNTN1/NF155 vs. CNTN1/NF155+Kif	<0.0001
CNTN1/NF155 vs. CNTN1+Kif/NF155	<0.0001
CNTN1/NF155 vs. CNTN1mut+Kif/NF155	0.9911
CNTN1+Kif/NF155+Kif vs. CNTN1/NF155+Kif	<0.0001
CNTN1+Kif/NF155+Kif vs. CNTN1+Kif/NF155	<0.0001
CNTN1+Kif/NF155+Kif vs. CNTN1mut+Kif/NF155	0.998
CNTN1/NF155+Kif vs. CNTN1+Kif/NF155	0.0099
CNTN1/NF155+Kif vs. CNTN1mut+Kif/NF155	<0.0001
CNTN1+Kif/NF155 vs. CNTN1mut+Kif/NF155	<0.0001
