# Supplementary material for: Structural insights into the contactin 1 – neurofascin 155 adhesion complex
Source: Nat Commun. 2022 Nov 3;13:6607. doi: 10.1038/s41467-022-34302-9 (PMC9633819; doi:10.1038/s41467-022-34302-9)
Supplement: Supplementary file 3 — Description of additional Supplementary File [file 41467_2022_34302_MOESM3_ESM.pdf]

Descriptions of Additional Supplementary Files

Supplementary movie 1: Structure of the contactin 1Ig1-6 – neurofascin 155Ig1-6 complex in cartoon representation with contactin 1Ig1-6 colored orange and neurofascin 155Ig1-6 colored blue. Glycan moieties are shown in stick representation and domains are labelled at the start.

Supplementary movie 2: Structure of the neurofascin 155Ig1-6 homodimer in cartoon representation with one molecule colored green and the other colored blue. Glycan moieties are shown in stick representation and domains are labelled at the start.

Supplementary movie 3: Structure of contactin 1Ig1-6 in cartoon representation colored orange. Glycan moieties are shown in stick representation and domains are labelled at the start.
