# Supplementary material for: Structural insights into the contactin 1 – neurofascin 155 adhesion complex
Source: Nat Commun. 2022 Nov 3;13:6607. doi: 10.1038/s41467-022-34302-9 (PMC9633819; doi:10.1038/s41467-022-34302-9)
Supplement: Supplementary file 7 — Reporting Summary [file 41467_2022_34302_MOESM7_ESM.pdf]

nature portfolio

Corresponding author(s): Bert Janssen

Last updated by author(s): Oct 11, 2022

Reporting Summary

Nature Portfolio wishes to improve the reproducibility of the work that we publish. This form provides structure for consistency and transparency in reporting. For further information on Nature Portfolio policies, see our Editorial Policies and the Editorial Policy Checklist.

Statistics

For all statistical analyses, confirm that the following items are present in the figure legend, table legend, main text, or Methods section.

n/a	Confirmed	
☐	☒	The exact sample size (*n*) for each experimental group/condition, given as a discrete number and unit of measurement
☐	☒	A statement on whether measurements were taken from distinct samples or whether the same sample was measured repeatedly
☐	☒	The statistical test(s) used AND whether they are one- or two-sided<br>*Only common tests should be described solely by name; describe more complex techniques in the Methods section.*
☐	☒	A description of all covariates tested
☐	☒	A description of any assumptions or corrections, such as tests of normality and adjustment for multiple comparisons
☐	☒	A full description of the statistical parameters including central tendency (e.g. means) or other basic estimates (e.g. regression coefficient) AND variation (e.g. standard deviation) or associated estimates of uncertainty (e.g. confidence intervals)
☐	☒	For null hypothesis testing, the test statistic (e.g. *F*, *t*, *r*) with confidence intervals, effect sizes, degrees of freedom and *P* value noted<br>*Give P values as exact values whenever suitable.*
☒	☐	For Bayesian analysis, information on the choice of priors and Markov chain Monte Carlo settings
☒	☐	For hierarchical and complex designs, identification of the appropriate level for tests and full reporting of outcomes
☒	☐	Estimates of effect sizes (e.g. Cohen's *d*, Pearson's *r*), indicating how they were calculated

Our web collection on statistics for biologists contains articles on many of the points above.

Software and code

Policy information about availability of computer code

Data collection	Integrated data were obtained for neurofascin 155 Ig1-6 and contactin 1 Ig1-6 datasets, from the xia2 dials (version 1.14.2) diamond beamline data auto processing pipeline, and further processed in AIMLESS version 0.7.4.
Data analysis	Unmerged and unscaled data were obtained from the xia2 dials (version 1.14.2) diamond beamline data auto processing pipeline for two isomorphic contactin 1Ig1-6 – neurofascin 155 Ig1-6 datasets collected in succession from the same crystal. These data were used to produce a combined unmerged and unscaled dataset using POINTLESS (version 1.12.2). Anisotropic cut-off, merging and scaling of the combined dataset was performed by the STARANISO webserver (version 2.3.52). All structures were solved by molecular replacement using PHASER (version 2.8.2). Models were generated in PHYRE2 (version 2.0). Structure refinement of unliganded datasets was performed using PHENIX (version 1.19.2). Manual model building was done in COOT (version 0.96). Structure refinement of the contactin 1Ig1-6 – neurofascin 155Ig1-6 dataset was initially performed using REFMAC (version 5.8.0267). MOLPROBITY (version 4.4) was used for structure validation. Electrostatic surface properties at pH 7.4 were obtained using the PDB2PQR (version 3.1.0) and APBS (version 3.0) webservers. Conservation analyses were performed using CONSURF (version 2019). Figures were generated with PyMol (version 2.4), and the ILLUSTRATE webserver (version 2019). Sequence alignment of L1 mouse paralogues highlighting architecture was prepared using ESPript webserver (version 3.0).<br>Collected chromatograms and MALS data were analyzed and processed using ASTRA6 software (version 6.1) (Wyatt).<br>SPR data was analyzed using SPRINTX (version 1.11) (IBIS Technologies) and PRISM (Graphpad) (version 9.0.2).<br>SAXS data reduction was performed automatically using the DAWN pipeline (2.27.0). Data was analyzed in PRIMUS (3.0.2).<br>AUC data was analyzed with SEDFIT (16-1c). Predicted scattering, fit to experimental scattering data, and Rg of the models were calculated using the FoXS webserver (version unknown).<br>The cluster size, co-cluster ratio, and clustering index were averaged per image, and data from three independent experiments (5 images per experiment, 15 images total per condition) were analysed using Python 3 (version 3) and Seaborn (version 0.11) statistical data visualization.

For the comparison of multiple groups, we used a one-way ANOVA followed by a Tukey's multiple comparison test using GraphPad Prism version 9 (GraphPad Software, San Diego, California USA version 9.0.2).

For manuscripts utilizing custom algorithms or software that are central to the research but not yet described in published literature, software must be made available to editors and reviewers. We strongly encourage code deposition in a community repository (e.g. GitHub). See the Nature Portfolio guidelines for submitting code & software for further information.

Data

Policy information about availability of data

All manuscripts must include a data availability statement. This statement should provide the following information, where applicable:

- Accession codes, unique identifiers, or web links for publicly available datasets
- A description of any restrictions on data availability
- For clinical datasets or third party data, please ensure that the statement adheres to our policy

Coordinates and structure factors for contactin 1Ig1-6 – neurofascin 155Ig1-6, neurofascin 155Ig1-6, contactin 1Ig1-6 have been deposited in the Protein Data Bank with accession numbers 7OL4 [http://doi.org/10.2210/pdb7OL4/pdb] (contactin 1Ig1-6 – neurofascin 155Ig1-6 complex), 7OK5 [http://doi.org/10.2210/pdb7OK5/pdb] (neurofascin 155Ig1-6), and 7OL2 [http://doi.org/10.2210/pdb7OL2/pdb] (contactin 1Ig1-6), respectively. All SAXS data have been deposited at the small angle scattering databank (SASBDB) with the accession codes: SASDL66 [www.sasbdb.org/data/SASDL66] (neurofascin 155Ig1-6 HM 37.2 µM), SASDL76 [www.sasbdb.org/data/SASDL76] (neurofascin 155Ig1-6 HM 73.5 µM), SASDL86 [www.sasbdb.org/data/SASDL86] (neurofascin 155Ig1-6 HM 19.7 µM), SASDL96 [www.sasbdb.org/data/SASDL96] (neurofascin 155Ig1-6 HM 9.5 µM), SASDLA6 [www.sasbdb.org/data/SASDLA6] (neurofascin 155Ig1-6 HM 5.1 µM), SASDLB6 [www.sasbdb.org/data/SASDLB6] (neurofascin 155Ig1-6 HM 2.7 µM), SASDLC6 [www.sasbdb.org/data/SASDLC6] (neurofascin 155Ig1-6 HM 1.3 µM) (neurofascin 155Ig1-6 HM), SASDLD6 [www.sasbdb.org/data/SASDLD6] (neurofascin 155Ig1-6 Thr216Ala HM 109.3 µM), SASDLE6 [www.sasbdb.org/data/SASDLE6] (neurofascin 155Ig1-6 Thr216Ala HM 21.9 µM), SASDLF6 [www.sasbdb.org/data/SASDLF6] (neurofascin 155Ig1-6 Thr216Ala HM 5.5 µM), SASDLG6 [www.sasbdb.org/data/SASDLG6] (neurofascin 155Ig1-6 Thr216Ala HM 1.1 µM) (neurofascin 155Ig1-6 Thr216Ala HM), SASDLH6 [www.sasbdb.org/data/SASDLH6] (contactin 1Ig1-6 CG 24.6 µM), SASDLJ6 [www.sasbdb.org/data/SASDLJ6] (contactin 1Ig1-6 CG 12.3 µM), SASDLK6 [www.sasbdb.org/data/SASDLK6] (contactin 1Ig1-6 CG 6.2 µM), SASDLL6 [www.sasbdb.org/data/SASDLL6] (contactin 1Ig1-6 CG 3.1 µM) (contactin 1Ig1-6 CG), SASDLM6 [www.sasbdb.org/data/SASDLM6] (contactin 1Ig1-6 HM 21.8 µM), SASDLN6 [www.sasbdb.org/data/SASDLN6] (contactin 1Ig1-6 HM 10.9 µM), SASDLP6 [www.sasbdb.org/data/SASDLP6] (contactin 1Ig1-6 HM 5.5 µM), SASDLQ6 [www.sasbdb.org/data/SASDLQ6] (contactin 1Ig1-6 HM 2.7 µM) (contactin 1Ig1-6 HM), SASDLR6 [www.sasbdb.org/data/SASDLR6] (contactin 1fe CG 20 µM), SASDLS6 [www.sasbdb.org/data/SASDLS6] (contactin 1fe CG 10 µM), SASDLT6 [www.sasbdb.org/data/SASDLT6] (contactin 1fe CG 5 µM), SASDLU6 [www.sasbdb.org/data/SASDLU6] (contactin 1fe CG 2.5 µM) (contactin 1fe CG), SASDLV6 [www.sasbdb.org/data/SASDLV6] (contactin 1fe HM 20.5 µM), SASDLW6 [www.sasbdb.org/data/SASDLW6] (contactin 1fe HM 11.1 µM), SASDLX6 [www.sasbdb.org/data/SASDLX6] (contactin 1fe HM 5.5 µM), SASDLY6 [www.sasbdb.org/data/SASDLY6] (contactin 1fe HM 2.4 µM) (contactin 1fe HM). Source data are provided with this paper.

Human research participants

Policy information about studies involving human research participants and Sex and Gender in Research.

Reporting on sex and gender	n.a.
Population characteristics	n.a.
Recruitment	n.a.
Ethics oversight	n.a.

Note that full information on the approval of the study protocol must also be provided in the manuscript.

Field-specific reporting

Please select the one below that is the best fit for your research. If you are not sure, read the appropriate sections before making your selection.

☒ Life sciences ☐ Behavioural & social sciences ☐ Ecological, evolutionary & environmental sciences

For a reference copy of the document with all sections, see nature.com/documents/nr-reporting-summary-flat.pdf

Life sciences study design

All studies must disclose on these points even when the disclosure is negative.

Sample size	Sample size for each replicate in the cell assay was 15 and chosen prior to the experiment. In our experiments, N = 3 for biological repeats is accepted in the field for drawing conclusions about cell clustering. We pre-determined taking 5 images per biological repeat to ensure at least hundreds of cells were available for the analysis.
Data exclusions	no data was excluded in the cellular assays
Replication	Experimental assays were performed at least in three independent replicates. All replications of the cellular assays were succesful.
Randomization	Large starting populations of cells were randomly distributed to different conditions.

Blinding	The analysis steps in the cellular assays were performed blindly. The other steps were not preformed blindly. We have applied a uniform set of techniques for the cellular assays. Image acquisition and analysis was standardized and uniformly applied. Thus further blinding was not necessary for our studies.

Reporting for specific materials, systems and methods

We require information from authors about some types of materials, experimental systems and methods used in many studies. Here, indicate whether each material, system or method listed is relevant to your study. If you are not sure if a list item applies to your research, read the appropriate section before selecting a response.

Materials & experimental systems

n/a	Involved in the study
☒	☐ Antibodies
☐	☒ Eukaryotic cell lines
☒	☐ Palaeontology and archaeology
☒	☐ Animals and other organisms
☒	☐ Clinical data
☒	☐ Dual use research of concern

Methods

n/a	Involved in the study
☒	☐ ChIP-seq
☒	☐ Flow cytometry
☒	☐ MRI-based neuroimaging

Eukaryotic cell lines

Policy information about cell lines and Sex and Gender in Research

Cell line source(s)	Suspension HEK293-E and HEK293-ES: U-Protein Express; suspension K562: American Type Culture Collection (CCL-243)
Authentication	Cells were not authenticated
Mycoplasma contamination	Cell lines were not tested for mycoplasma contamination
Commonly misidentified lines (See ICLAC register)	No commonly misidentified cell lines were used in this study.
